# Alport syndrome caused by a *COL4A5* deletion and exonization of an adjacent *AluY*

**DOI:** 10.1002/mgg3.89

**Published:** 2014-05-28

**Authors:** Kandai Nozu, Kazumoto Iijima, Yasufumi Ohtsuka, Xue Jun Fu, Hiroshi Kaito, Koichi Nakanishi, Igor Vorechovsky

**Affiliations:** 1 Department of Pediatrics, Kobe University Graduate School of MedicineKobe, Japan; 2 Department of Pediatrics, Faculty of Medicine, Saga UniversitySaga, Japan; 3 Department of Pediatrics, Wakayama Medical UniversityWakayama, Japan; 4 Faculty of Medicine, University of SouthamptonSouthampton, U.K

**Keywords:** *Alu*, collagen, exonization, intron, RNA processing

## Abstract

Mutation-induced activation of splice sites in intronic repetitive sequences has contributed significantly to the evolution of exon–intron structure and genetic disease. Such events have been associated with mutations within transposable elements, most frequently in mutation hot-spots of *Alu*s. Here, we report a case of *Alu* exonization resulting from a 367-nt genomic *COL4A5* deletion that did not encompass any recognizable transposed element, leading to the Alport syndrome. The deletion brought to proximity the 5′ splice site of *COL4A5* exon 33 and a cryptic 3′ splice site in an antisense *AluY* copy in intron 32. The fusion exon was depleted of purines and purine-rich splicing enhancers, but had low levels of intramolecular secondary structure, was flanked by short introns and had strong 5′ and *Alu*-derived 3′ splice sites, apparently compensating poor composition and context of the new exon. This case demonstrates that *Alu* splice sites can be activated by outlying deletions, highlighting *Alu* versatility in shaping the exon–intron organization and expanding the spectrum of mutational mechanisms that introduce repetitive sequences in mRNAs.

The mutation was found in a 14-year-old boy who developed macrohematuria at the age of 4 months. He was diagnosed with the Alport syndrome 14 months later following a confirmatory renal biopsy that showed a typical lamellation of the glomerular basement membrane and the absence of type IV collagen *α*5 chain by immunohistochemistry (data not shown) carried out as described (Oka et al. 2014). The patient developed mental retardation and autism; his severe proteinuria eventually culminated in renal failure at the age of 10 and he underwent preemptive renal transplantation using a kidney from his father. He did not show any detectable hearing loss or ocular abnormalities. His mother had hematuria and mild proteinuria since early childhood.

The disease-causing deletion was found by PCR amplifications of patient's DNA across exon 33, revealing a smaller fragment in the patient and in his heterozygous mother (Fig. 1A, left panel). DNA sequencing of the new fragment showed a 367-nt deletion (*COL4A5* c.2768-230_c.2904del367 at Xq22.3) encompassing most of the 150-nt exon 33 (Fig. 1B and C). Amplicons of cDNA samples reverse transcribed from blood or urine RNA also showed a fragment with a slightly greater mobility (Fig. 1A, right panel). Sequencing of the cDNA fragment revealed the inclusion of a new exon of 141 nt, which contained a premature termination codon in an *AluY-*derived sequence of intron 32 (Fig. 1B and D). The adjacent deleted sequence was devoid of any transposed elements, as determined by the most sensitive option of the RepeatMasker (http://www.repeatmasker.org/cgi-bin/WEBRepeatMasker, version 4.0.5), yet the deletion was capable of activating a distant cryptic 3′ splice site of the new exon 128-nt upstream of the deletion breakpoint in the left arm of the *AluY* copy. Thus, the fusion exon was composed of an *Alu*-derived sequence of intron 33, 15-nt linker between the *AluY* and the deletion breakpoint, and the 3′ end of exon 33 (Figs. 1D, S1 and S2).

**Figure 1:**
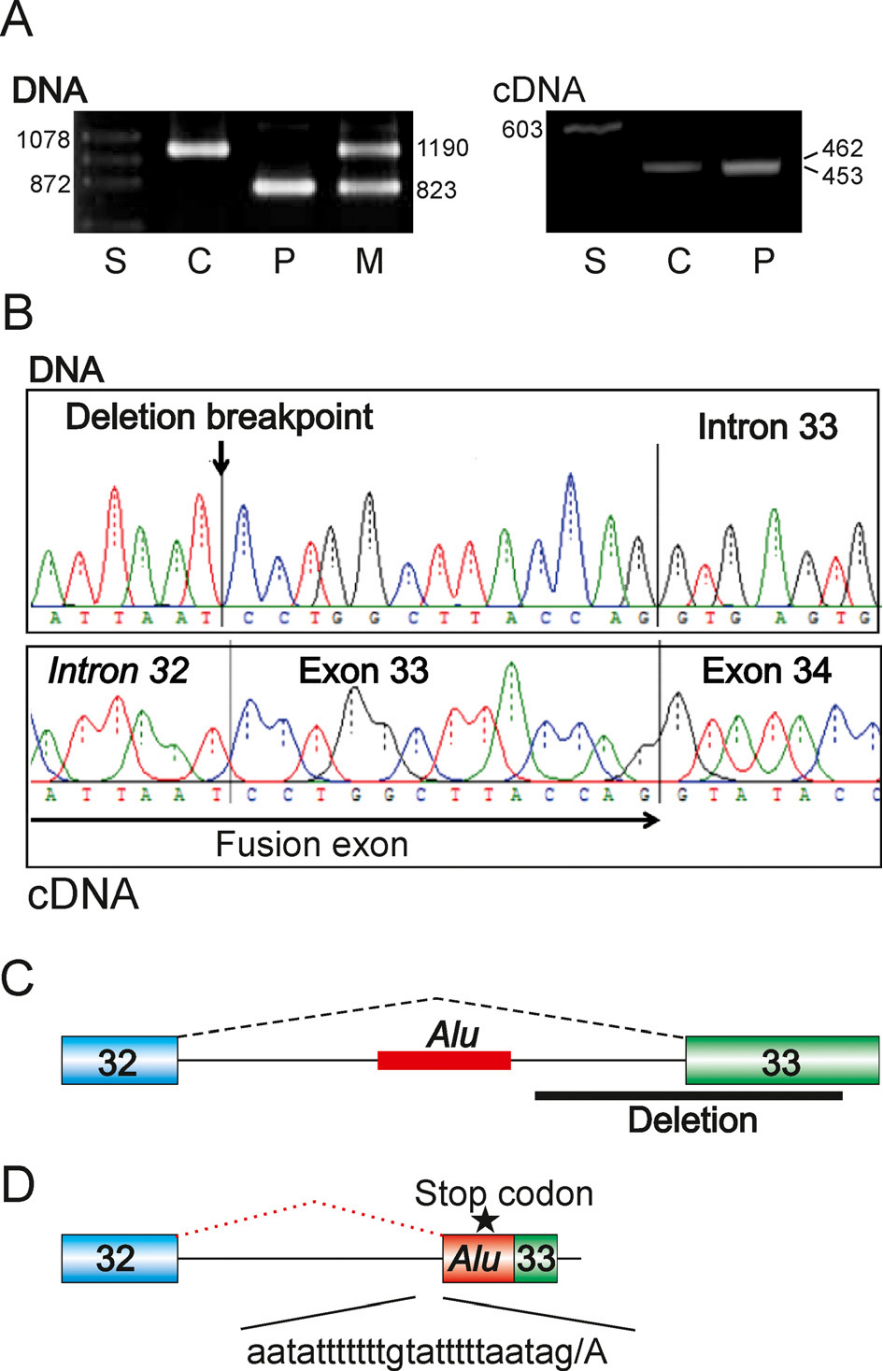
Deletion-induced exonization of *AluY* leading to Alport syndrome. (A) PCR amplifications of DNA (left panel) and cDNA (right panel) samples from a control (C), patient (P) and his mother (M). S, size marker; fragment sizes are shown in nts. DNA was amplified by primers 5′-AGTTTTCTGGTTGACATCTTA and 5′-ATAAGTCACTTTTCATGCTAT; cDNA was amplified by primers 5′-CAACCTGGTTTACATGGAAT and 5′-TCCAGGCAAACCCTGATAACC. (B) Sequence chromatogram of patient's DNA (upper panel) and cDNA (lower panel). (C, D) Schematic representation of the genomic deletion (C) and *AluY* exonization (D). Exons are shown as boxes, introns as horizontal lines, canonical (black) and aberrant (red) splicing by dotted lines above the primary transcripts. Sequence of the new 3′ splice is shown at the bottom, forward slash denotes the new intron–exon boundary. Location of the stop codon is shown by an asterisk.

Although cryptic exons derived from transposed elements causing genetic disease contain on average more splicing enhancers and less silencers than average human exons (Vorechovsky 2010), the new fusion exon was rich in pyrimidines and in splicing silencers and was depleted of purine-rich enhancers, the most potent exon recognition motifs. For example, the density of exon identity elements (Zhang et al. 2008) was only 68% of the average exon density and less than a half of the density calculated for the deleted exon counterpart (Table 1). This was also reflected in a lower predicted stability across the *Alu* portion of the new exon, consistent with a higher single-strandedness observed for exonizing *Alu*s than for nonexonizing *Alu*s (Schwartz et al. 2009). However, the 3′ splice site of the new exon was stronger than that of exon 33, most likely compensating the unfavorable nucleotide composition of the new exon (Table 1) in the context of a strong 5′ splice site (score 95.04). Finally, the inclusion of the new exon in the *COL4A5* mRNA (Fig. 1A) may have been assisted by shortening of the intron preceding exon 33 by a half and by a relatively short downstream intron, because long introns tend to associate with nonexonizing rather than exonizing *Alu*s (Schwartz et al. 2009).

**Table 1:** Comparison of sequence features of the new *Alu* exon and exon 33

	%T	%C	%A	%G	EIE density^1^	3′ splice site score^2^	Free energy^3^
Deleted exon 33	18.0	24.0	26.7	31.3	660	75.96	−0.37
New *Alu* exon	25.5	29.1	22.0	23.4	324	82.18	−0.27

1 Density of exon identity elements (EIEs) (Zhang et al. 2008) was computed as described (Divina et al. 2009).

2 Shapiro–Senapathy score was calculated by an online tool at http://ibis.tau.ac.il/ssat/SpliceSiteFrame.htm.

3 kcal/mol and nt at 37°C.

Multiple sequence alignments of available primate orthologs coupled with evolutionary reconstruction of the insertion event (Krull et al. 2005) showed the presence of this *Alu* in Old World monkeys and the same 3′ splice-site consensus of the new exon across species (TAG/A). This *Alu* element was absent in New World monkeys (*Platyrrhini*), indicating that the insertion took place ∼40–25 million years ago and that the 5' breakpoint (/) of the 367-deletion in the patient was in the target-site duplication sequence GGATTAAGCATTAAT/TTTTTT. Thus, the ancient *Alu* insertion was a prerequisite not only for the exonization event but also for the genomic deletion found in the family.

*Alu* exonization has facilitated gene regulation through alternative splicing during primate evolution and contributed to the expansion of proteomic interactions in humans (Makalowski et al. 1994; Sorek et al. 2002; Krull et al. 2005), however, only a very limited number of exonization mechanisms has been described, both for existing exons (Lev-Maor et al. 2003; Sorek et al. 2004) and disease-associated events (Meili et al. 2009; Vorechovsky 2010). This case demonstrates that a disease-causing *Alu* exonization can result from deletions not involving any transposed elements and reveals key sequence features promoting activation of the *Alu* exon with a poor splicing enhancer/silencer ratio, expanding the range of mutational mechanisms that introduce the most common human repeats in the mRNA.

## Supporting Information

**Figure S1.** Alignment of antisense *COL4A5* intronic *AluYm1* in patient ID45 and the *AluYm1* consensus. v, transversions; i, transitions. The 3′ splice-site AG is in red. **Figure S2.** The genomic sequence of *COL4A5* across the fusion exon. Exons are in upper case, introns are in lower case. Deletion is highlighted in gray; sequence of the new fusion exon is in italics; target-site duplications are underlined.
