# Supplementary material for: Alport syndrome caused by a COL4A5 deletion and exonization of an adjacent AluY
Source: Mol Genet Genomic Med. 2014 May 28;2(5):451–3. doi: 10.1002/mgg3.89 (PMC4190880; doi:10.1002/mgg3.89)
Supplement: Supplementary file 1 [file mgg30002-0451-SD1.docx]

**Supplemental data**

**Figures S1 Alignment of antisense *COL4A5* intronic *AluYm1* in patient ID45 and the *AluYm1* consensus**

**Legend:** v, transversions, i, transitions. The 3’ splice site AG is in red.

ID45 TTTTTTTGTTTGTCTTTTGAGACGGAGTCTCGCTCTGTTGCCCAGGCTGG 286

v v i i

AluYm1 TTTTTTTTTTTTTTTTTTGAGACGGAGTCTCGCTCTGTCGCCCAGGCTGG 250

ID45 AGTGCAATGGTGCGATCTCAGCTCACTCCAAATTCCATCTCCTGGGTTCA 336

i i i v ii ii i

AluYm1 AGTGCAGTGGCGCGATCTCGGCTCACTGCAAGCTCCGCCTCCCGGGTTCA 200

ID45 CACTATTCTCCTGCCTCAGCCTCCCAAGTAGCTGGGAGTACAGGCGCCTG 386

i i i v i

AluYm1 CGCCATTCTCCTGCCTCAGCCTCCCGAGTAGCTGGGACTACAGGCGCCCG 150

ID45 CCACCACGCCCGGCTAATATTTTTTTGTATTTTTAATAGAGAAGGATTTT 436

--- i v iv

AluYm1 CCACCACGCCCGGCTAA---TTTTTTGTATTTTTAGTAGAGACGGGGTTT 103

ID45 CACCATGTTAGCAAGGATGGTCTCGATCTCCTGACCTCGTGATCTGCCCA 486

i v i i

AluYm1 CACCGTGTTAGCCAGGATGGTCTCGATCTCCTGACCTCGTGATCCGCCCG 53

ID45 CCTCAGCCTCCCAAAGTGCTGGGATTACAGGCTTGAGCCACCGTGCCCG 535

i i

AluYm1 CCTCGGCCTCCCAAAGTGCTGGGATTACAGGCTTGAGCCACCGCGCCCG 4

**Figure S2 The genomic sequence of *COL4A5* across the fusion exon**

**Legend:** Exons are in upper case, introns are in lower case. Deletion is highlighted in grey; sequence of the new fusion exon is in italics; target site duplications are underlined.

GTACCAAAGGTGAAATGGGTATGATGGGACCTCCAGGCCCACCAGGACCTTTGGGAATTCCTGGCAGGAGTGGTGTACCTGGTCTTAAAG

gtaataatcaaggtttgctgccagacgtatgtgagagggaaaattaaatatagctttatg

tcagtacagaatatttttgttgactgttttaaaatgagcaatggttacttgtgtttctat

gtaacatggcatttaaataggtcttgttttcaatttggttacgtgcttagtttggatacc

atgatacatggtaaatgcttagctcaaaataacaaaatataaggattaagcattaatttt

tttgtttgtcttttgagacggagtctcgctctgttgcccaggctggagtgcaatggtgcg

atctcagctcactccaaattccatctcctgggttcacactattctcctgcctcagcctcc

caagtagctgggagtacaggcgcctgccaccacgcccggctaatatttttttgtattttt

aatag/*agaaggattttcaccatgttagcaaggatggtctcgatctcctgacctcgtgatc*

*tgcccacctcagcctcccaaagtgctgggattacaggcttgagccaccgtgcccgtctgg*

*attaagcattaat*ttttttatagtcggcctctatcgttgtcaaggaattccagatggagg

gattgttatctgagtataaaaatgtgtatgcactcaagtaattcatagagtacatccaag

gtgcataaatgttttgagtggccagtggcctactcaactatgaaacatatcaataatctt

tatatgcattaatctttgatggataaaattgatatattgtgttttcacacacattgattt tagGTGATGATGGCTTGCAGGGTCAGCCAGGACTTCCTGGCCCTACAGGAGAAAAAGGTAGTAAAGGAGAGCCTGGCCTTCCAGGCCCTCCTGGACCAATGGATCCAAATCTTCTGGGCTCAAAAGGAGAGAAGGGGGAA*CCTGGCTTACCAG*

gtgagtgaatgaatttatttatgaatatttttcctgatatatctgaagtttaatttttaa
